# The larynx in cough

**DOI:** 10.1186/1745-9974-9-16

**Published:** 2013-06-03

**Authors:** Guri S Sandhu, Romana Kuchai

**Affiliations:** 1 Consultant Otolaryngologist, Imperial College London, London, UK

**Keywords:** Chronic, Cough, Larynx, Nose, Post-nasal drip, Gastro-esophageal reflux, Laryngopharyngeal reflux

## Abstract

About 40% of the population will experience chronic cough at some point during their lives and it tends to be more common in women (Thorax 58:901–7, 2003). Post-nasal drip (or *upper airway cough syndrome*), gastro-esophageal reflux disease and asthma are considered the most common causes. Yet only a small percentage of patients with these common conditions experience chronic cough. Also there is no agreed measure of post-nasal drip and controversy exists about the diagnosis of reflux above the upper esophageal sphincter (*laryngopharyngeal reflux*) based on observable changes to the larynx. The approach of the otolaryngologist is to consider the upper and lower airways as a continuum and that a common pathology can have an impact on all these anatomical sites.

A multidisciplinary approach is advocated, utilising the skills of the respiratory physician, otolaryngologist, gastroenterologist and speech pathologist.

## Introduction

Chronic cough is experience in approximately 40% of people at some stage in their lives and seems to affect women more commonly than men [1]. A cough is defined as a forced expiration against a closed glottis, which opens suddenly, with a characteristic sound and expulsion of secretions and foreign materials from the respiratory tract [2]. It can be voluntary or involuntary and often repetitive. The phases that form the reflex are:

– inhalation due to contraction of diaphragm and external intercostal muscles

– forced exhalation (internal intercostal and abdominal muscles) against a closed glottis

– trachealis contracts to narrow and make the trachea and principle bronchi rigid

– sudden forced release of air from the lower respiratory tract through an open glottis

– associated characteristic sound.

Acute cough is considered to last under three weeks; sub- acute cough is that which lasts three to eight weeks and chronic cough is defined as lasting longer than eight weeks. The principle function of the larynx is to protect the airway. This is achieved by abduction of the true and false cords, posterior deflection of the epiglottis and the larynx rising to lie below and behind the tongue base. All this happens during the pharyngeal phase of swallowing and is an involuntary reflex which prevents food and secretions entering the airway. The secondary function of the human larynx is speech. To achieve this complex form of communication the larynx in man lies lower in the neck than other mammals enabling better resonance (pharynx, nasal cavity, sinuses and mouth) and articulation (lips, tongue and teeth). This has exposed the larynx to a greater risk of aspiration and gastric reflux disorders.

### Physiology of the larynx in cough

In discussing the role of the larynx in cough we discuss it in its chronic persistent form. It remains a significant illness burden within the community, in particular, as part of the chronic cough syndrome. The cough reflex is initiated by the stimulation of sensory receptors in the larynx and lower respiratory tract which subsequently send signals to the brainstem. The central organisation of this is poorly understood however these receptors are known to lie within the sub-epithelial layer throughout the respiratory tract. The receptors are rapidly adapting myelinating fibres in the vagus nerve. Two types of afferent nerves consist of myelinated fibres and the non-myelinated fibres C-fibres with nerve endings within the lungs. The exact role of each is yet to be fully understood [3]. The stimulation of these C-fibres is thought to cause mast cell degranulation and subsequent oedema which itself may activate the adaptive receptors. The activation of C-fibre receptors within the respiratory tract releases sensory neuropeptides causing neurogenic inflammation. The central connections of the C-fibre receptors are thought to inhibit the cough reflex. A complex of interaction between C-fibres, rapidly adapting receptors, peripheral and central nervous systems are the reason for the sensitivity of the reflex. The relationship of the physiology and the clinical basis for the reflex is yet poorly understood.

The superior layngeal nerve conveys most of the afferent fibres. Aspiration of a small particle of food may activate a violent cough episode and both mechanical and chemical irritants may stimulate the cough reflex. Although trans-laryngeal pressure receptors are thought to act as drive receptors during the respiratory cycle, they are not thought to participate in the cough reflex.

Chronic cough often starts with a ‘cold’ or ‘flu’ like illness and the cough persists beyond the acute phase of the illness. It is possible that one or more pre-existing factors such as gastroesophageal reflux or post-nasal drip, were previously not sufficient to initiate a cough, now help to perpetuate it in combination with the trauma to the larynx from the physical act of coughing [4]. This prolonged airway inflammation, perpetuated by multiple aetiologies may explain why some authorities are now hypothesizing a *cough hypersensitivity syndrome*[5]. The concept of a hypersensitized larynx serves as a good model for advising patients on treatments.

It is clear that further research into the pathophysiology of the processes that interact and activate the cough reflex is very much needed to improve our understanding and thereby long-term management.

Otolaryngologists are usually referred patients with symptoms of cough to help establish if the cause is related to post nasal drip or gastroesophageal reflux disease and this can be a complex process as some may exhibit clear signs whilst other have none.

### Diagnosis

It is important to bear in mind the concept of ‘one airway, one disease’ which considers the upper and lower airway as a continuum where inflammation can be secondary to a common pathology. Inflammation can release histamine and induce cough by stimulating the respiratory tract. We describe the causes of chronic cough by sub-dividing it upon an anatomical basis, however, it must be kept within context of the single airway.

The most common causes of chronic cough are said to be Post Nasal Drip (PND), asthma related syndromes and gastroesophageal reflux disease (GERD) [6]. The Otolaryngologist, upon assessing a patient with chronic cough, will take a careful history and the examination will include endoscopy of the nasal cavity, larynx and pharynx. A chest X-ray will be requested, if not all ready done, and referral to a respiratory physician if history and findings dictate. Where there is suspicion of allergy, skin allergy tests will be performed and in patients with sinus symptoms, a computer tomography (CT scan) may be advisable. A gastroenterology referral is made either when there is frank and severe reflux or a definitive trial of medical therapy has failed to bring about symptom relief. Where there is concern about the safety of swallowing, or oesophageal dysmotility is suspected, then a Video Fluoroscopic Assessment of Swallowing is required but the sensitivity of this test is dependent entirely upon the skills and experience of the radiologist and swallowing therapist conducting it.

The initial assessment of cough may focus upon general and specific causes. This can at times be difficult due to the multifactorial nature of the symptoms. In the absence of pathology, a cough that fails to resolve spontaneously or in response to definitive medical treatment may be described as idiopathic, although some of these cases will be psychogenic. Some authorities have described the incidence of idiopathic cough to be as high as 31% [7] however, in the authors’ practice the incidence is actually much lower. Table 1 outlines the aetiologies of chronic cough seen in the ENT clinic.

**Table 1:** The causes of chronic cough seen in the ENT clinic

**Nasal**	**Laryngeal**	**Lower respiratory tract**	**Others**
Post-nasal drip	GERD/LPR	Asthma	Psychogenic
	Laryngeal dysfunction	Eosinophilic bronchitis	Idiopathic
	Laryngotracgeal stenosis	COPD	
	Swallowing disorders	Interstitial Lung Disease	
	Systemic Diseases	Foreign Body	
	Neurological		
	Drugs		
	OSAS		

### Nasal

#### Post-nasal drip

The nose acts as a filter, a humidifier and also warms the air that reaches the larynx and trachea. Conditions that lead to nasal obstruction bypass this nasal function. It is also entirely physiological for secretions from the nose (20–40 mls each day) to enter the pharynx, through the ciliary action of the nasal mucosa, and be swallowed. It is described as post-nasal drip when patients report having the sensation of mucus tracking down into the throat, having a nasal discharge or needing to clear the throat frequently. It may coincide with nasal congestion and discharge.

**Figure 1:**
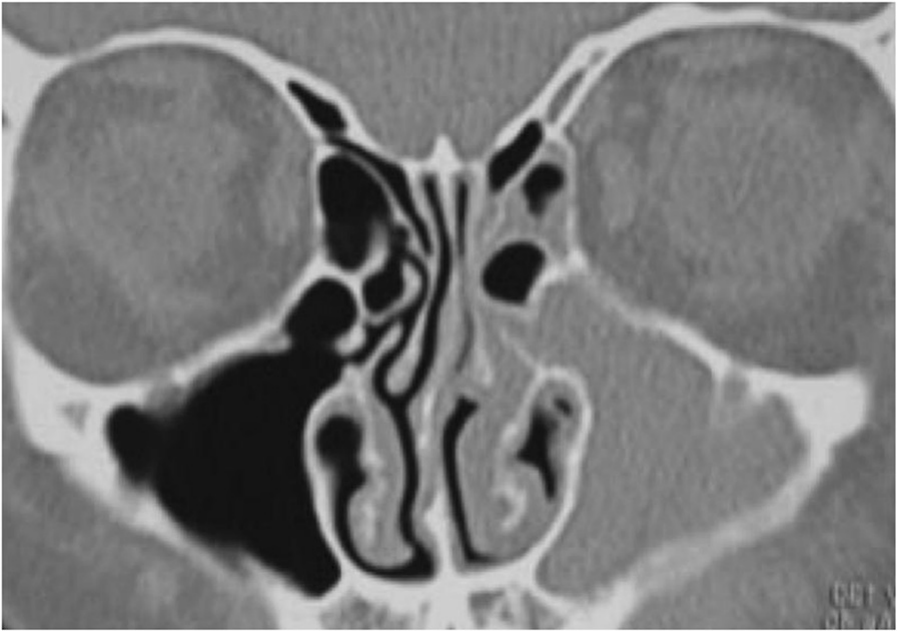
Coronal CT scan through the paranasal sinuses demonstrating extensive nasal polyposis.

**Figure 2:**
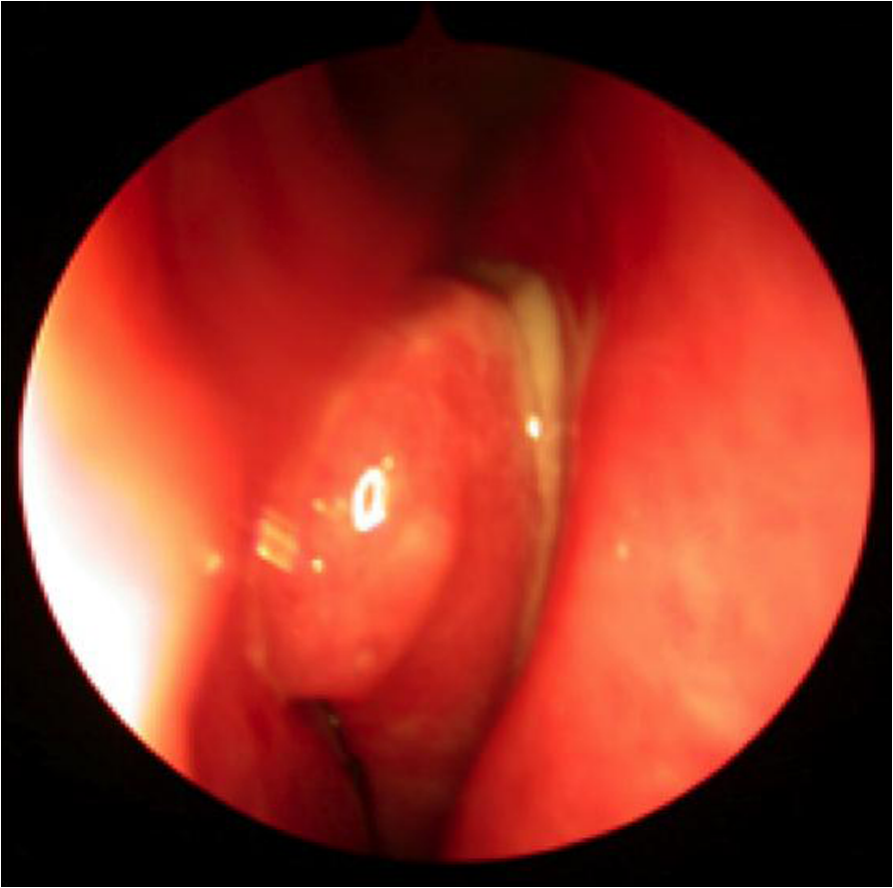
Endoscopic view of the nose demonstrating inflammation, mucopus and polypoid change.

Several rhinological conditions are associated with post nasal drip. These include allergic rhinitis, chronic rhinosinusitis and nasal polyps (Figure 1). Chronic rhinosinusitis (Figure 2) is recognised as the main cause of PND [8] and is defined as inflammation of the lining of the nose and paranasal sinuses, characterised by one or more of the following symptoms: nasal congestion, rhinorrhoea, sneezing, itching and hyposmia.

Over time there has been much debate as to whether post nasal drip syndrome is, in fact, a syndrome and its relationship with chronic cough is often questioned. It would seem sensible to think patients with chronic rhinosinusitis would complain of post nasal drip syndrome, however, only a small number also present with chronic cough [9].

There is, as yet, no objective method for evaluation of the symptoms and diagnosis follows retrospective treatment based upon a thorough clinical assessment. Guidelines from the American College of Chest Physicians have recommended using the term *upper airway cough syndrome* (UACS) instead of PND [10] to reflect that conditions causing postnasal drip, such as rhinitis, may have a similar coexisting effect on the larynx.

The treatment of post nasal drip is to initially treat the underlying cause. The recent Allergic Rhinitis and its impact on Asthma guidelines [11] recommend first-line treatment with intra-nasal corticosteroids. Antihistamines are to be started if symptoms of sneezing, itchy eyes and clear nasal discharge are evident. Topical corticosteroids sprays are the initial treatment of choice in chronic rhinosinusitis, with or without nasal polyposis, by The EPOS Guidelines [8]. It is advisable to apply an initial three month course of topical corticosteroids with an essential review at six months. In moderately symptomatic cases topical steroid drops may be applied with a review at three month intervals. At present there is no clear evidence, however, of the impact of topical corticosteroids upon cough. Gawchik et al. [12] in the only randomised control trial showed topical steroids were effective upon chronic cough, associated with post nasal drip syndrome, following a two to eight week course. The aims of this trial as a multi-centre double-blind were to specifically assess the effectiveness of Mometasone furoate nasal spray(MFNS) upon seasonal allergic rhinitis associated cough (SAR). Patients were treated with 200 mcg daily for fourteen days. The daytime cough group showed a significant improvement whilst only a trend in favour of treatment was shown with the night-time cough group. The study revealed MFNS is effective in the management of daytime cough associated with SAR.

### Laryngeal

#### Laryngopharyngeal infections

**Figure 3:**
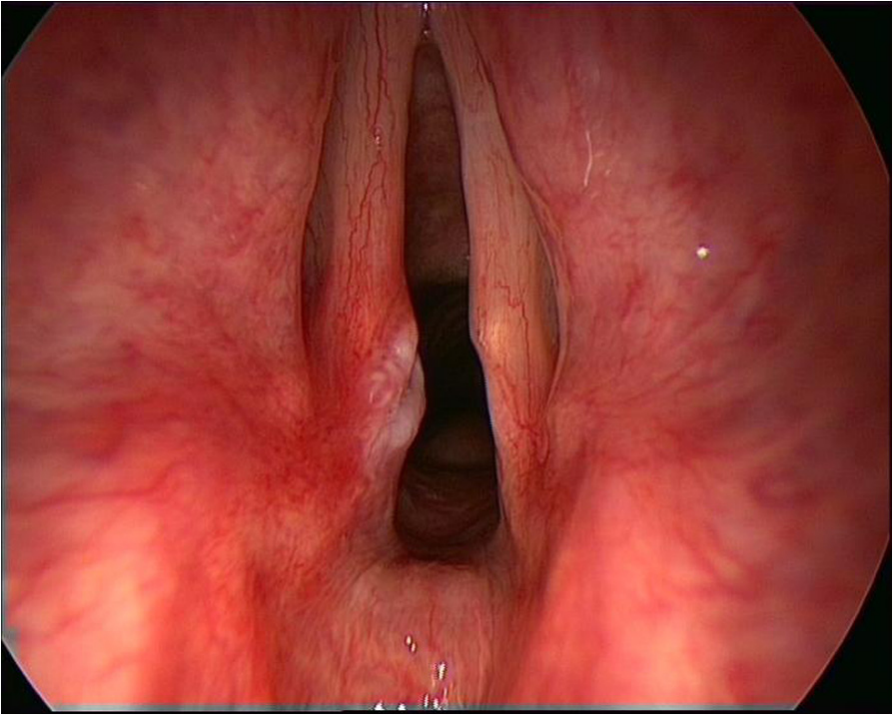
View of vocal cords demonstrating an injury to the post vocal cord in a patient with chronic cough.

Upper respiratory tract infections are most commonly viral in nature and are associated with inflammation of the larynx and pharynx. Treatment must be conservative with voice rest, steam inhalation, adequate hydration. The majority of these infections resolve spontaneously. Some of these infections may be primarily or become bacterial in nature and should be treated with a course of antibiotics. Interestingly it is common for a chronic cough condition to start with such a respiratory tract infection. This would suggest that there may have been a pre-existing, low level, process producing laryngeal inflammation and this infection has been enough to ‘tip the balance’. It is also possible that coughing itself is enough to cause sufficient laryngeal trauma to sustain chronic inflammation and laryngeal hyper-reactivity (Figure 3).

Some authorities believe that some cases of chronic cough may be due to a post-viral laryngeal sensory neuropathy [13]. Cranial nerves are known to be affected by inflammatory neuropathic processes as seen in Bell’s palsy and trigeminal neuralgia. These conditions can also result in altered sensory and motor nerve function [14]. Sensory neuropathic cough is thought to be analogous to the lowered threshold to stimuli seen in cases of trigeminal or post-herpetic neuralgias. It is speculated that with the vagus nerve this is mediated as a ‘bogus tickle’ [15] that leads to uncontrollable coughing. Many of these cases have been reported to have responded to Amitryptilline (10 mg nocte for at least 21 days). Amitryptilline may lower the sensory threshold for the afferent nerve endings but may also be having a psychotropic affect [15]. Other drugs being considered to treat sensory neuropathic cough include gabapentin and pregabalin but more research needs to be done.

#### Laryngopharyngeal reflux

Gastroesophageal reflux disease (GERD) is considered to be the cause of chronic cough in up to 40% of patients [16]. The diagnosis of GERD is based on well defined symptom scoring and 24 hour pH testing. Those that argue for different diagnostic criteria for laryngopharyngeal reflux (LPR), do so, on the basis that the larynx is very poorly protected against even transient reflux episodes and that the reflux material also contains proteolytic enzymes and bile salts, both of which can cause laryngeal irritation.

Symptoms of reflux include [17]:

– Sensation of lump in the throat

– Frequent clearing of the throat

– Hoarseness

– Dysphagia

– Feeling of mucus in the back of the throat

– Heartburn/dyspepsia.

**Figure 4:**
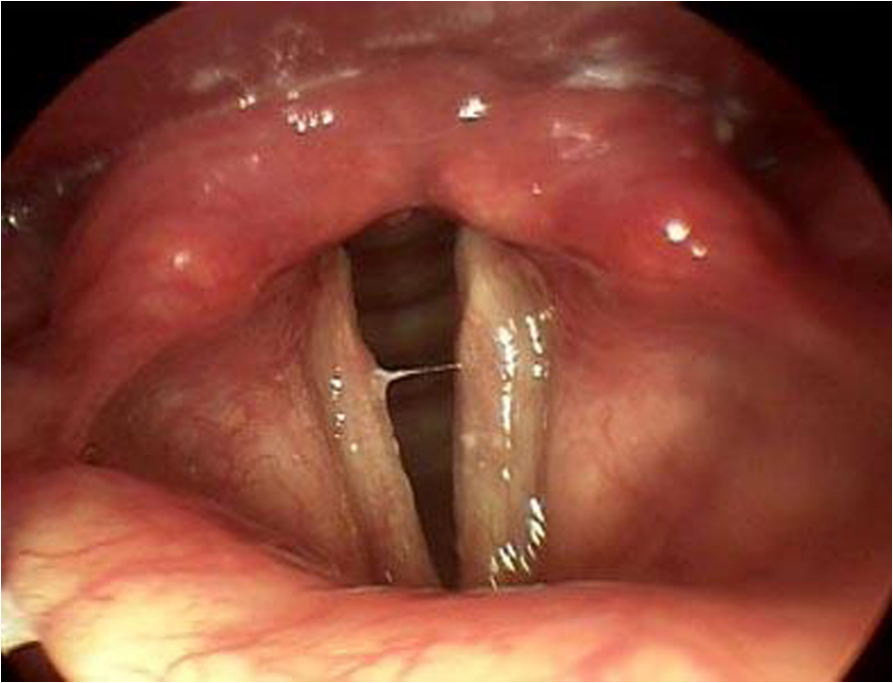
Manifestations of laryngopharyngeal reflux, posterior laryngeal swelling and obliteration of laryngeal ventricles.

Some ENT surgeons believe that laryngeal erythema and LPR are related but this is a non-specific finding that is considerably dependent on the examination technique. Belafsky et al. [18] confirmed LPR by dual probe pH monitoring in a group of patients and found the most common sign was posterior laryngeal hypertrophy (in 85%). Laryngeal ventricle obliteration was seen in 80% of these patients (Figure 4).

Standard 24 hour pH studies report reflux events where the pH drops below pH 4 for at least 6 seconds and for greater than 5% off the time of monitoring. The gold standard for diagnosis of LPR remains multichannel intraluminal impedance manometry (IMM). A catheter placed in the oesophagus measures the change in electrical impedance during the upward passage of a fluid. It enables measurement of the pH and also the height and clearance of the reflux.

It is generally an accepted practice to treat with a trial of proton- pump inhibitors when suspecting reflux as a cause for chronic cough and reserving further investigations if medical therapy fails. Aggressive treatment (twice daily) with a proton pump inhibitor may be necessary for a period of two to three months to reverse the effects of LPR. An alginate such as Gaviscon Advance should be used after the evening meal to deal with the non-acidic components of the refluxate [17]. Lifestyle changes are considered crucial and complementary in the long-term management of patients. This often includes weight-loss and dietary changes such as limiting caffeine consumption, early evening meals within 3 hours of sleep and elevation of the head of the bed.

Severe cases of gastroesophageal reflux- related cough, not responding to medical treatment, may be referred for anti-reflux surgery such as Nisssen’s fundoplication. It is more difficult to convince the surgical community to undertake this procedure for patients diagnosed with LPR.

### Disorders of swallowing

Any disturbance of normal swallowing may result in aspiration and chronic cough. However poor clearance of secretions in the hypopharynx and dysmotility may have similar affects. Over 2% of the elderly population suffer with age-related swallowing problems (presbyphagia) and these may be compounded by poor dentition, increased pharyngeal transit time and neurological issues. Even in the absence of co-morbidities it is recognised that the oesophageal wall becomes stiffer [19] and there is a decrease in the oesophageal ganglion count [20] with increasing age. Table 2 summaries known causes of disorders of swallowing.

**Table 2:** Disorders leading to swallowing problems

**Neurological**	**Autoimmune**	**Head and neck conditions**	**Others**
Stroke	Systemic Sclerosis	Caustic Ingestion	Presbyphagia
Amyotrophic Lateral	Systemic Lupus	Head and Neck/Neurologocal	Pharyngeal Pouch
Sclerosis	Erythematosis	Tumours	
Parkinson’s Disease	Dermatomyositis	Post Surgery or Radiotherapy	
Multiple Sclerosis	Mucosal Pemphigoid		
Muscular Dystrophy	Epidermolysis Bullosa		
Myasthenia Gravis	Sjogren’s Syndrome		
	Rheumatoid Arthritis		

Videofluoroscopy is a useful investigation in assessing swallowing disorders and provides an excellent dynamic assessment of all phases of swallowing with reasonable anatomical detail. Its disadvantage, however, is the obvious exposure to radiation, its lack of ability to test sensitivity and of course the logistics of its organization. A fibreoptic endoscopic evaluation of swallowing (FEES) is an alternative more cost-effective investigation with no radiation exposure. It is dependent upon the availability of a nasendocope and is unable to formally evaluate the function of the cricopharyngeus.

A pharyngeal pouch must be excluded when considering the laryngopharyngeal causes of chronic cough and can be identified by a contrast study, such as a barium swallow. It is management depends upon the severity of symptoms. Endoscopic stapling is a common modality of surgical treatment, however, a large or recurrent pouch may necessitate an open procedure.

#### Laryngeal dysfunction

Chronic cough is increasingly understood to be associated with laryngeal symptoms. Hypersensitivity of the larynx due to sensory hyper-responsiveness characterises this phenomenon. The effect upon the larynx in augmenting the cough reflex with agents such as capsaicin have helped establish the fact that the cough reflex indeed the only motor reflex of sensory activation. Although vocal cord disorder (VCD) is associated with chronic cough, it is important to understand the distinction.

Vocal fold dysfunction is considered to be a result of paradoxical vocal fold movement (PVCM) and results in reduced inspiratory airflow [21]. Compared with the cough alone and healthy group, they also reported an overlap in symptomatologies of chronic cough and VCD. In a randomised control trial it was established that speech therapy, normally provided for vocal fold dysfunction, also proved therapeutic in the treatment of chronic cough. Ryan et al. [22], in their study of 25 patients with persistent chronic cough, observed paradoxical vocal fold movement in 56% of subjects. Extra-thoracic airway hyper-responsiveness was significantly increased in the cough with PVCM group.

Paradoxical vocal fold movement causes glottis closure and upper airway narrowing. This narrowing, thereby results in symptoms of cough, shortness of breath and wheeze and consequently may be difficult to distinguish from asthma. An enhanced glottis stop reflex in chronic cough patients has been shown to be precipitated when the larynx is exposed to chemical inhaled irritants as shown by Prudon et al. [23]. This mechanism results in PVCM however may be one of many other stimulants such as chronic inflammation of the larynx, gastro-oesophageal reflux disease and possibly even chronic rhinosinusitis.

### Laryngospasm

Laryngospasm is defined as a spasm of the vocal folds which temporarily interrupts breathing. It may last up to 30 seconds and if there is loss of consciousness the cords relax. It is important when managing these patients that they are reassured that they will not die. Any underlying or associated conditions such as allergy or reflux must be treated and the patient is counselled to ‘sniff’ in order they may break the spasm. Intra-laryngeal Botox has been used within the course of their management (Additional file 1, based on the principle author’s practice and experience) in those that develop uncontrolled frequency of symptoms impacting upon their daily lives [24].

### Laryngotracheal stenosis

Laryngotracheal stenosis usually presents with a difficulty in breathing and is associated with poor exercise tolerance but can be associated with a chronic cough. Its presentation is often insidious and is sometimes misdiagnosed as asthma. The causes of laryngotracheal stenosis are congenital or acquired. In the adult population 50% are related to ventilation on the intensive care unit.

The management of laryngotracheal stenosis is dependent upon its formal assessment to establish its size, location and any associated factors. This usually necessitates direct visualisation of the airway under a general anaesthetic with endoscopic laser laryngotracheoplasty and balloon dilatation. Regular review with more than one treatment episode may be required in the long-term management of these cases and those, that remain symptomatic, may be considered for an open procedure such as laryngotracheal reconstruction.

### Systemic conditions

The most commonly encountered conditions are sarcoidosis and Wegener’s Granulomatosis. The former most commonly impacts upon the supraglottis whilst the latter within the subglottis and tracheobronchial tree. Symptoms may vary from alteration in the quality of voice to a significant difficulty in breathing depending upon the size and location of the disease in each case. A chronic cough is often associated with these conditions when they involve the airway. A multi-disciplinary approach to treatment is an established practice with surgical intervention complementing medical therapy.

### Head and neck conditions

Benign or malignant lesions of the larynx may be associated with a cough. The management of these is beyond the scope of this text but may include endoscopic or open surgical procedures and in the case of non-resectable malignancies also radiotherapy.

### Psychogenic cough

Much neurobiological research has been undertaken investigating the role of higher brain areas in cough, however, there is little systematic behavioural research on the role of psychological factors. Van den Bergh et al. [25], in a review of the psychology of cough, suggest there is significant evidence supporting the role of several basic psychological processes on the urge to cough and cough behaviour. Attention, cognition, emotion, learning and social factors all are thought to impact upon the processes determining the relation between central cortical mechanisms and the psychological sub-functions they subserve. Overall evidence is fairly sparse, however, sufficiently suggestive to necessitate further systematic research in this field.

## Conclusions

Chronic cough is a common but complex symptom that requires careful thought and consideration within the context of each case. A multi-disciplinary approach, no doubt, is the key to its management and this is where an Otolaryngologist needs to understand the conditions that independently contribute to the symptoms. Direct endoscopic examination of the nose, larynx and pharynx enable the identification of signs that would otherwise be missed. The management very much depends upon the cause and treatment often remains fairly preliminary with its success being determined by symptomatic response.

### Advice to a patient with chronic cough

– Carry water – take a sip of cold water to suppress the urge to cough. ‘Humm’ or gently throat clear until you get to the water as this causes less trauma to the larynx than a cough.

– Steam inhalation – ten minutes two to three times a day will be soothing to the larynx add menthol crystals if preferred

– sleep with head of bed elevated

– lose weight (if advice is appropriate)

– dietary changes to minimise gastric reflux

avoid allergens or cough triggers.

### Treatments

– Proton pump inhibitor twice daily for 2–3 months if evidence of GERD or LPR

– Alginate (Gaviscon Advance) after lunch and evening meal

– Treat the Nose and PND with nasal steroids (add antihistamines if allergy suspected or confirmed))

– Stop ACE inhibitors (but related affects may not reverse for 2–3 months) and use alternative

– BOTOX into larynx as last resort. Helps laryngospasm but also enforces laryngeal rest from trauma of coughing.

Further work with randomised controlled trials analysing the symptoms and treatment pathways is essential to improve management of chronic cough and outcomes that successfully improve the quality of life of patients should remain the primary objective.

## Abbreviations

PND: Post-nasal drip; UACS: Upper airway cough syndrome; GERD: Gastro-esophageal reflux disease; CT: Computer tomography; ACE: Angiotensin converting enzyme; OSAS: Obstructive sleep apnoea syndrome; COPD: Chronic obstructive pulmonary disease.

## Competing interests

The authors declare that they have no competing interests.

## Authors’ contributions

GS snd RK have a specific clinical interest in the role of the larynx in cough and have performed a comprehensive literature search. Both authors have drafted, read and approved the final manuscript.

## Supplementary Material

Additional file 1 **A guide to the management of laryngospasm **[24]**.** Click here for file
