# Supplementary figures and images for: The larynx in cough
Source: Cough. 2013 Jun 3;9:16. doi: 10.1186/1745-9974-9-16 (PMC3704827; doi:10.1186/1745-9974-9-16)

## Slide 1
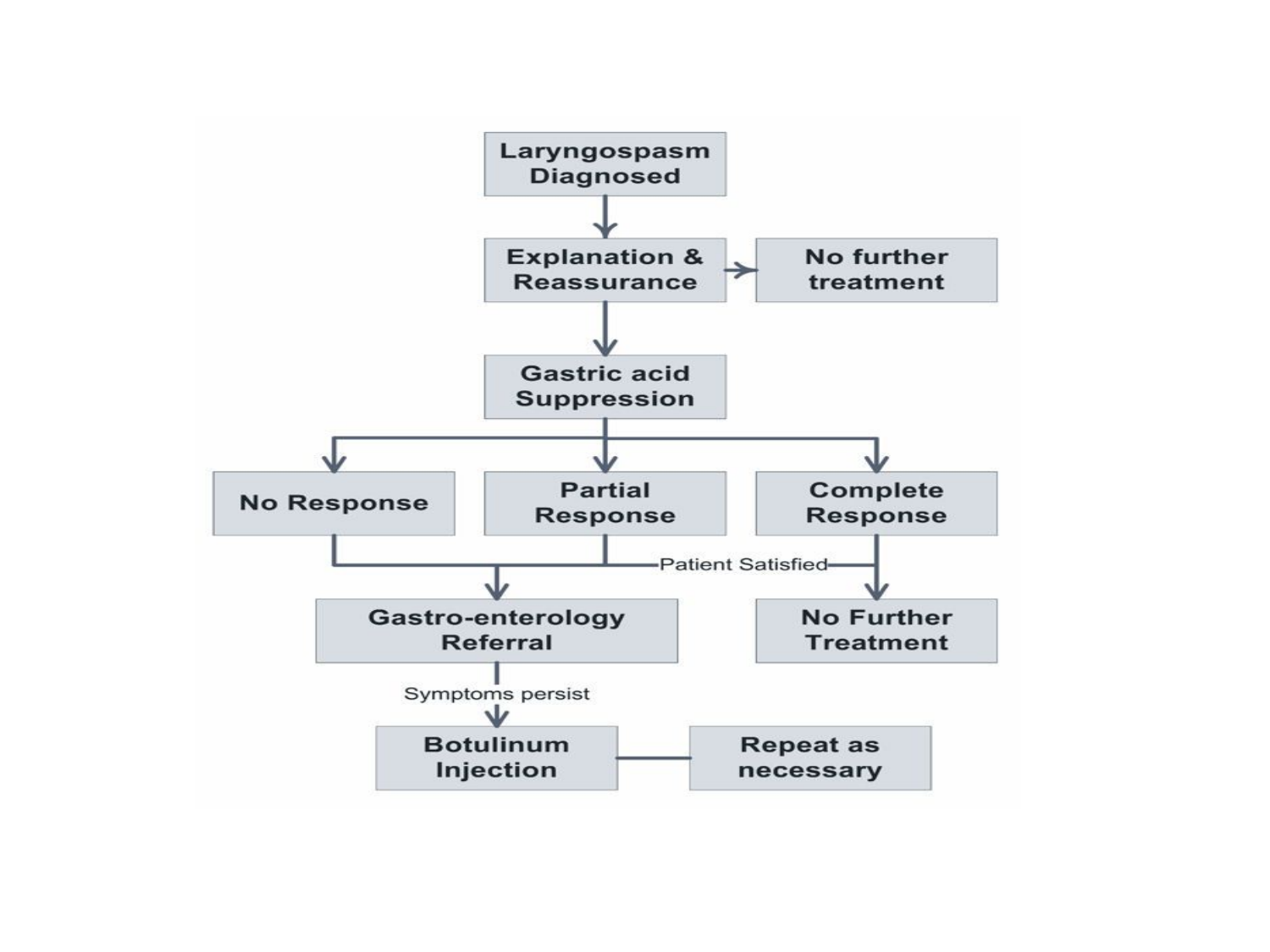

Supplement: Additional file 1 — A guide to the management of laryngospasm [24]. [file 1745-9974-9-16-S1.pptx]
